# Treatment of spinal cord injury with mesenchymal stem cells

**DOI:** 10.1186/s13578-020-00475-3

**Published:** 2020-09-22

**Authors:** Ling Ling Liau, Qi Hao Looi, Wui Chuen Chia, Thayaalini Subramaniam, Min Hwei Ng, Jia Xian Law

**Affiliations:** 1 Department of Physiology, Faculty of Medicine, Universiti Kebangsaan Malaysia Medical Centre, 56000 Kuala Lumpur, Malaysia; 2 Ming Medical Services Sdn. Bhd., Pusat Perdagangan Dana 1, 47301 Petaling Jaya, Selangor Malaysia; 3 Centre for Tissue Engineering and Regenerative Medicine, Faculty of Medicine, Universiti Kebangsaan Malaysia Medical Centre, JalanYaacob Latif, Bandar Tun Razak, 56000 Kuala Lumpur, Malaysia

**Keywords:** Spinal cord injury, Stem cell therapy, Mesenchymal stem cells, Nerve regeneration

## Abstract

**Background:**

Spinal cord injury (SCI) is the damage to the spinal cord that can lead to temporary or permanent loss of function due to injury to the nerve. The SCI patients are often associated with poor quality of life.

**Results:**

This review discusses the current status of mesenchymal stem cell (MSC) therapy for SCI, criteria to considering for the application of MSC therapy and novel biological therapies that can be applied together with MSCs to enhance its efficacy. Bone marrow-derived MSCs (BMSCs), umbilical cord-derived MSCs (UC-MSCs) and adipose tissue-derived MSCs (ADSCs) have been trialed for the treatment of SCI. Application of MSCs may minimize secondary injury to the spinal cord and protect the neural elements that survived the initial mechanical insult by suppressing the inflammation. Additionally, MSCs have been shown to differentiate into neuron-like cells and stimulate neural stem cell proliferation to rebuild the damaged nerve tissue.

**Conclusion:**

These characteristics are crucial for the restoration of spinal cord function upon SCI as damaged cord has limited regenerative capacity and it is also something that cannot be achieved by pharmacological and physiotherapy interventions. New biological therapies including stem cell secretome therapy, immunotherapy and scaffolds can be combined with MSC therapy to enhance its therapeutic effects.

## Background

The spinal cord connects most of the periphery nervous system to the brain and is protected by a vertebral column that is divided into multiple segments. These segments are grouped into 4 major regions, C1–C8 cervical, T1–T12 thoracic, L1–L5 lumbar and S1–S5 sacral vertebrae. Spinal nerves protruded from different segments of vertebral column innervate different body regions. The cervical nerves control the motor and sensory functions of the head, neck, shoulder, arm and hand. The thoracic nerves control the motor and sensory functions of the chest and abdominal walls. The lumbar nerves innervate the hip and leg and the sacral nerves supply the genitals and lower digestive tract. Within the vertebral column, the spinal cord is covered and protected by the meninges (dura mater, arachnoid mater and pia mater) and cerebrospinal fluid (in subarachnoid space between the arachnoid and pia mater) [1–3]. Apart from serving as a conduit for the transmission of sensory input to the brain and motor output to the effector tissues, the spinal cord is also responsible for the production of spinal reflex that protects the body from harmful stimuli [4].

A spinal cord injury (SCI) is damage to the spinal cord that can lead to temporary or permanent changes to the cord’s normal motor, sensory and autonomic function, resulting in calamitous neurological deficiency and disability [5]. The prevalence and incidence of SCI are ranging from 8 to 906 cases per million people according to the countries and regions and is more common in males below 30 years old [6]. SCI can affect most of the bodily functions including breathing, bowel and bladder function, hormone release, and sexual function, due to the loss of connection between the brain and the peripheral nervous system. Although the severity of SCI and resulting disability differ from individual to individual, nonetheless, it will significantly affect the patient quality of life. After injury, regeneration of spinal cord axons is very limited due to the low growth capacity of neurons, presence of inhibitory factors in central nervous system myelin, the formation of glial scar, and lack of neurotrophic factors and nerve growth factors [7, 8]. Conventional treatment of SCI focuses on stabilizing the injured area via surgery, preventing secondary injury through pharmacological intervention, and rehabilitation to prevent loss of function and to help regain the loss functions [9, 10]. However, these treatments have limited success as they cannot stimulate spinal cord regeneration.

**Fig. 1:**
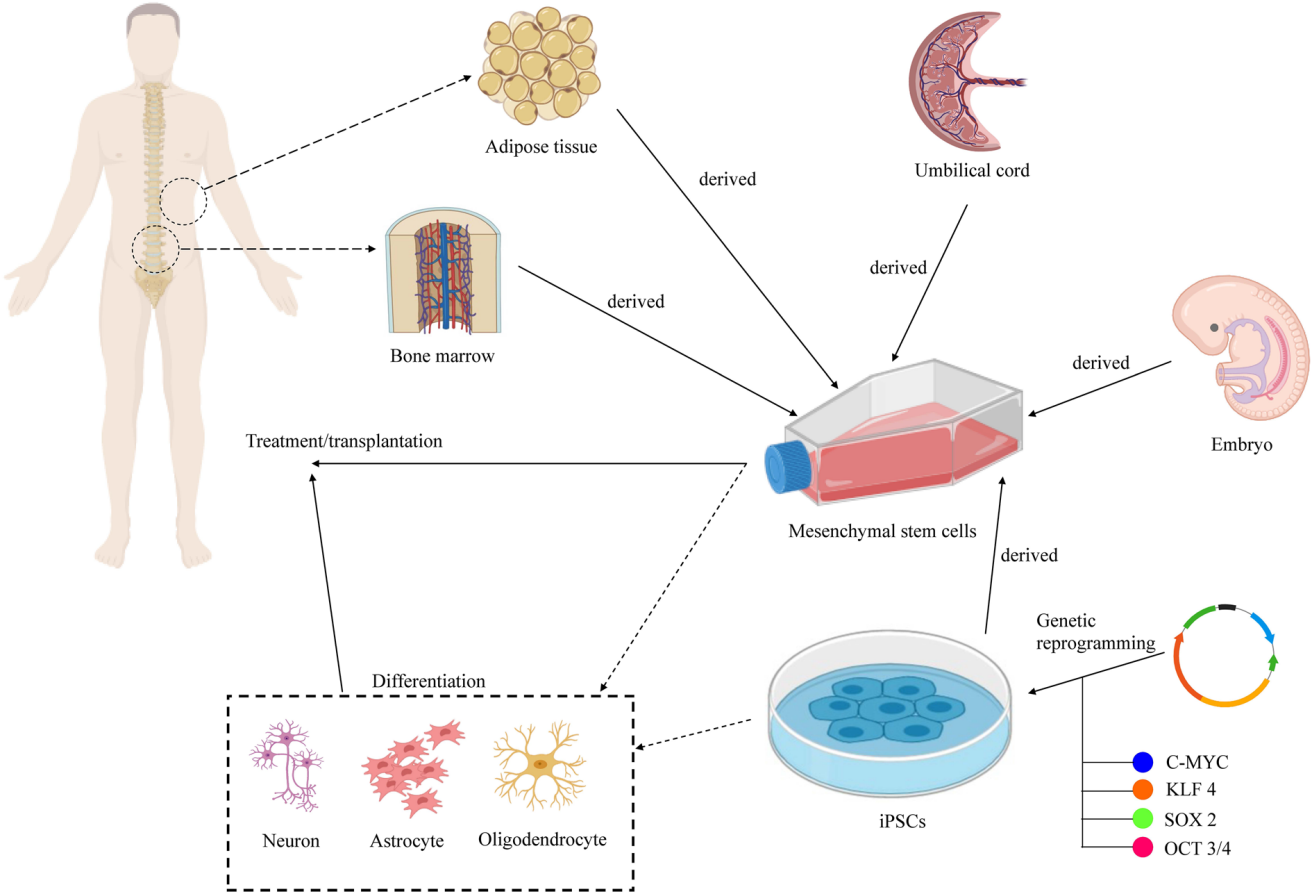
Stem cell therapy for SCI. Human embryonic stem cells, bone marrow-derived mesenchymal stem cells, adipose-derived mesenchymal stem cells, umbilical cord-derived mesenchymal stem cells and induced pluripotent stem cells can be used for the treatment of SCI

Several types of stem cells have been tested or being tested clinically for the treatment of SCI, including MSCs, ESC-derived oligodendrocyte precursor cells, fetal-derived neural stem cells, and central nervous system stem cells [11]. Figure 1 shows the applications of stem cells in the treatment of SCI. Thus far, most of the trials used MSCs isolated from bone marrow (BMSCs), umbilical cord (UC-MSCs) and adipose tissue (ADSCs) to treat SCI. MSCs were used to treat SCI as the cells can suppress the inflammation to limit the secondary injury, secrete paracrine factors that protect the remaining axons and promote axonal regeneration, and differentiate into nerve cells to replace the damaged cells [12, 13]. Both autologous and allogeneic MSCs have been used as the cells can evade rejection by the host immune system [14, 15].

In this review, we discussed the current status of MSC therapy for SCI focusing on the clinical trial results, the criteria to consider when applying the MSC therapy and the novel biological therapies that can be used together with MSC therapy to enhances its therapeutic potential.

## Spinal cord injury

SCI is classified as tetraplegia and paraplegia depending on the spinal cord segment affected. Tetraplegia refers to the injury at the cervical segment that causes loss of bodily control below the neck while paraplegia involves damage to the thoracic segment and the segments below that may affect all or part of the trunk, pelvis and legs. Paraplegics have full control of the arms and hands [16]. Based on the severity, SCI can be categorized as ‘complete’ if the control over the area below the affected area is loss totally and as ‘incomplete’ when some of the sensation, muscle function, and bodily functions are retained [17].

**Fig. 2:**
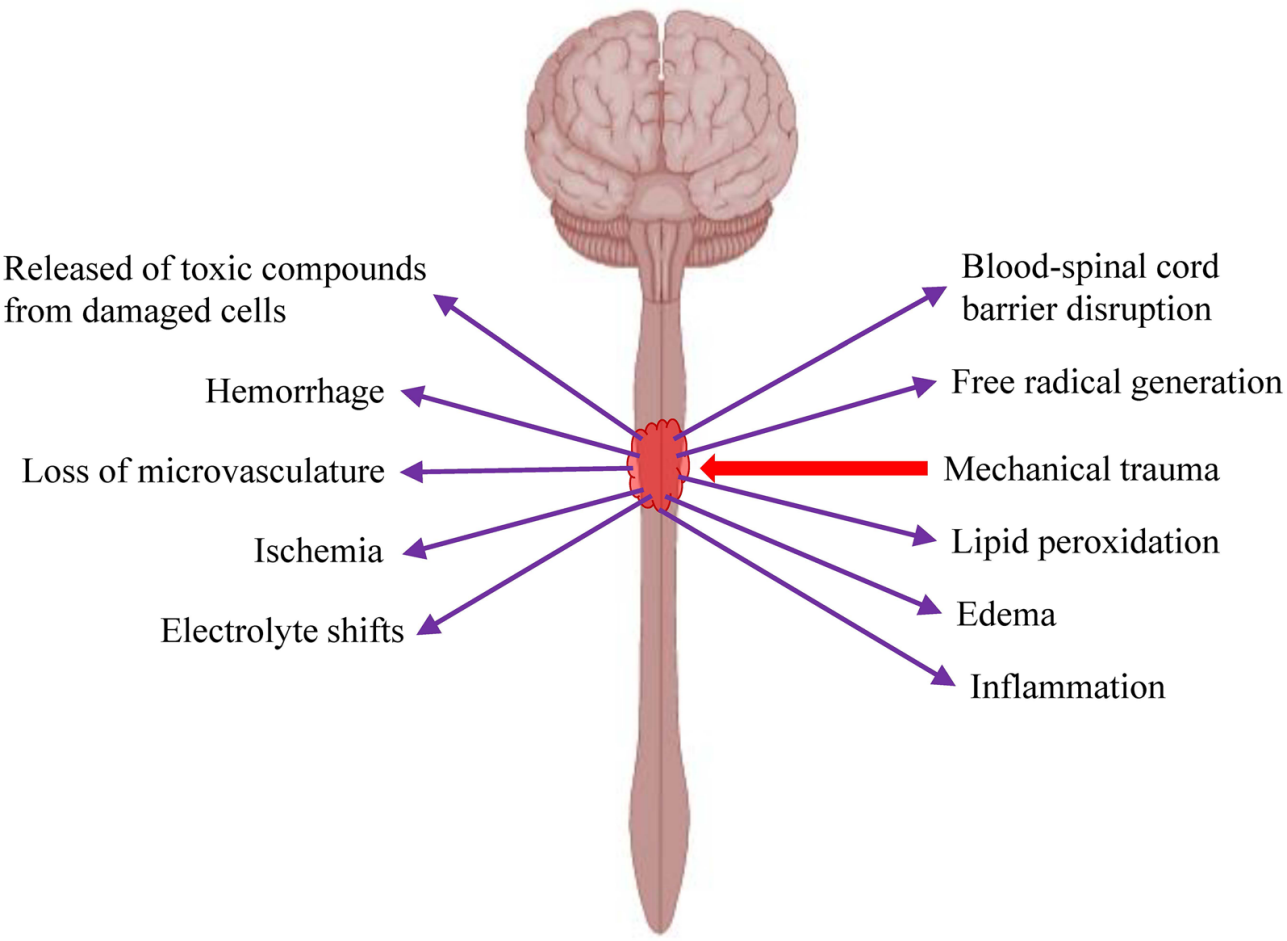
Changes to the injured spinal cord at the molecular and cellular level. Mechanical trauma to the spinal cord will cause bleeding, oedema, disruption in blood supply, inflammation, disruption of blood-spinal cord barrier, electrolyte shifts, oxidative stress, lipid peroxidation, cell necrosis and release of toxic substances by the damaged cells

Traumatic SCI can be divided into 2 phases, i.e. irreversible primary injury that happens immediately at the moment of injury and secondary injury that kicks in within minutes after the primary injury [18]. Mechanical injury to the spinal cord will lead to disruption of blood-spinal cord barrier (BSCB), cord hemorrhage, swelling, ischemia, inflammation, loss of microvasculature, electrolyte shifts, free radical generation, lipid peroxidation and release of toxic compounds from damaged neurons and glial cells that trigger the progressive secondary injury (Fig. 2). The intense inflammation and toxin released at the injury site will trigger the apoptosis of neurons and glial cells. Besides, the proinflammatory cytokines and chemokines will stimulate the differentiation of neural stem/progenitor cells into astroglia, leading to the transition from the inflammation phase to scar formation [19]. Deposition of the extracellular matrix (ECM) components (e.g. glial fibrillary acidic protein (GFAP), nestin and vimentin), and accumulation and activation of glial cells contribute to the formation of glial scar around the periphery of the lesion. Fibroblasts and Schwann cells will migrate to the center of the lesion and secrete the ECM proteins such as collagen, fibronectin and laminin, leading to fibrotic scarring. In the early phase, the formation of glial scar plays an important role in containing the injury area, limiting the inflammation, clearing the debris, regeneration of blood–brain barrier, preventing the spread of toxic compounds to the surrounding tissue and production of neurotrophins. However, at the later phase, glial scar and fibrotic scar, as well as the presence of axonal growth inhibitors, prevented the neuronal regeneration [20, 21]. Besides, neurons in the spinal cord have low regeneration and proliferation potential.

Spinal cord damage can be reduced by preventing, attenuating and reversing secondary injury. Many studies look into interventions targeting secondary injury with the hope of preserving and improving spinal cord neurological functions [16, 22–24]. Pathophysiology of traumatic SCI has been excellently summarized by Ahuja et al. that divided traumatic SCI temporally into the acute (0–48 h), subacute (2–14 days), intermediate (2 weeks to 6 months) and chronic (> 6 months) based on its pathophysiology [2]. Currently, ASIA (American Spinal Injury Association) Impairment Scale (AIS) is the gold standard used to classify SCI. AIS graded the SCI from A to E based on the sensory and motor function preserved. Table 1 indicates the classification of AIS grading [25].

**Table 1:** Classification of AIS grading

Grade	Characterizations
A	Complete loss of control of the body areas by the lowest sacral nerves
B	Retention of feeling but not movement below the level of injury
C	More than half the key muscles below the level of injury can move, but not against the gravity
D	More than half of the muscle below the level of injury can move against the gravity
E	Normal sensory and motor function for patient with history of SCI

## Mesenchymal stem cell therapy for spinal cord injury

**Fig. 3:**
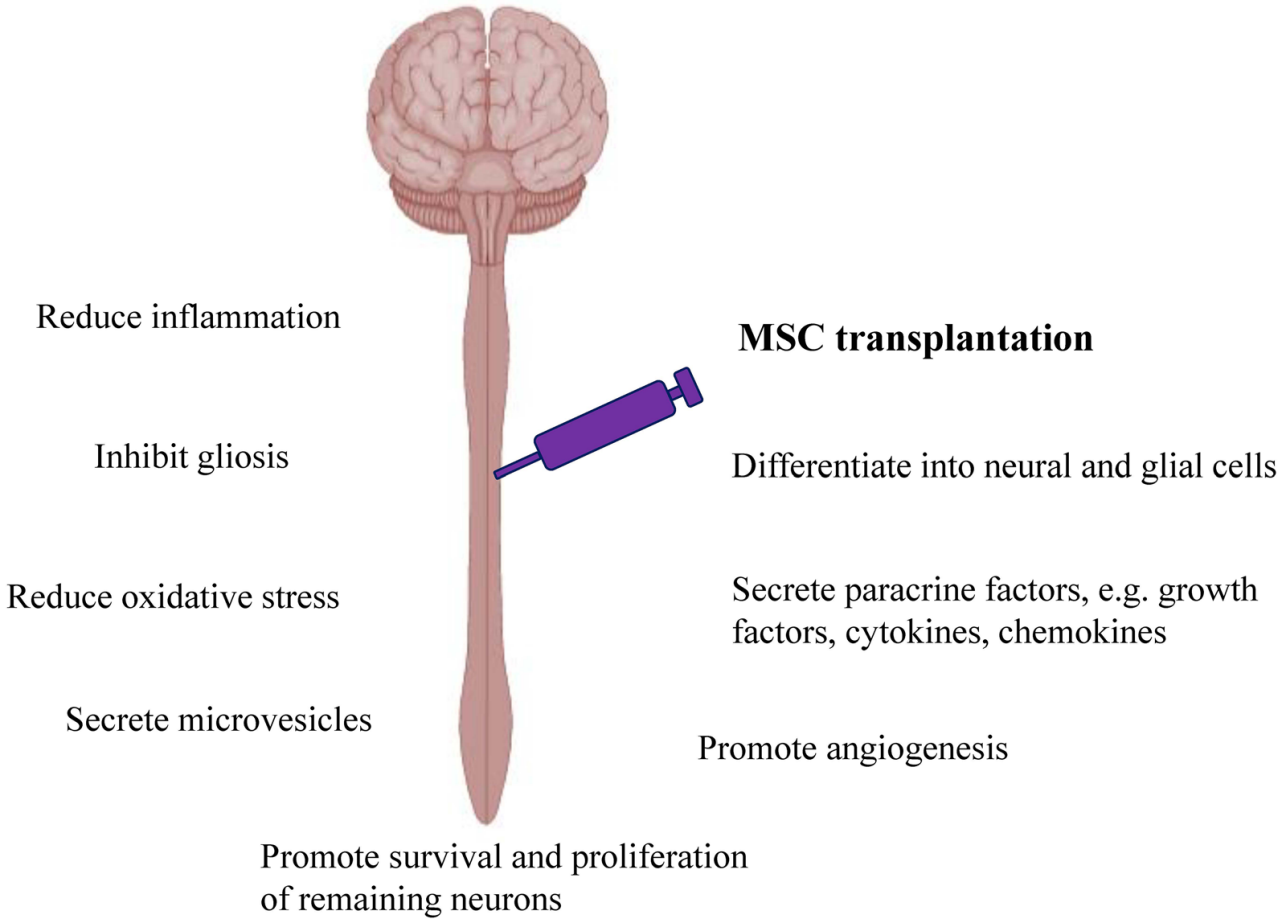
Mechanisms of action of MSCs in ameliorate SCI. MSC transplantation promotes the spinal cord regeneration by differentiating into neural and glial cells, secrete paracrine factors and microvesicles, reduce inflammation and oxidative stress, promote survival of remaining neurons and angiogenesis as well as inhibit gliosis

Traditionally, the regenerative capacity of MSCs was thought to be related to its plasticity to differentiate into neural and glial cells [26, 27]. However, recent studies suggested that the therapeutic effect is mostly exerted by their paracrine activity as MSCs have been found to secrete a broad range of bioactive molecules [28]. MSCs secrete VEGF, HGF, IGF-I, stanniocalcin-1, TGF-β, and GM-CSF that promote the survival of damaged neurons and oligodendrocytes [29, 30]. Together with PIGF, MCP-1, bFGF and IL-6, these factors also stimulate angiogenesis [31]. Proliferation and regeneration of the remaining neurons are stimulated by the GDNF, BDNF, and NGF secreted by the MSCs [32]. MSCs exert its immunomodulatory effects via cell-to-cell contact and secretion of IL-10, TGF-β, PGE-2, galectin-1, indolamine 2,3 dioxygenase (IDO) and HLA-G [30, 33–35]. By controlling the inflammation, MSCs reduce the damages done to the remaining and surrounding unaffected tissues. MSCs also can inhibit gliosis, thus improving the ECM environment for better neurite growth [36]. Besides, MSCs have antioxidant property and can stimulate cells to produce antioxidant enzymes [37, 38]. Apart from the paracrine factors, MSCs also have been reported to exert its therapeutic effect via direct cell fusion, mitochondrial transfer and production of microvesicles [39]. Figure 3 summarizes the mechanisms of action of MSC therapy.

### Bone marrow-derived mesenchymal stem cells

BMSC is the most popular source of MSCs for the treatment of SCI with more than 20 clinical trials registered on the ClinicalTrials.gov database. Generally, none of the studies was able to improve the neurological function of all the recruited patients. The key findings of these clinical trials are summarized in Table 2.

**Table 2:** Clinical trials conducted using bone marrow-derived mesenchymal stem cells for the treatment of SCI

References	Acute, subactute or chronic	AIS grade	Number of patients	Sources of MSCs	Route of administration	Number of cells	Frequency of injection	Safety outcome	Efficacy outcome
[40]	Chronic	A and B	10	BM	Intrathecal	1.48 × 10^8^ BMSCs	3 (every 4 weeks)	No AE	Improvement in ASIA score, SEP and EMG
[41]	Subacute and chronic	A and B	10	BM	Intrathecal	1.48 × 10^8^ BMSCs	3 (every 4 weeks)	No AE	Long-term follow-up (30–40 months) showed that 3 patients with AIS grade B demonstrated improvement in motor power of the upper extremities, activities of daily living and positive electrophysiological changes. MRI showed reduction in cavity size and presence of fiber-like low signal intensity steaks
[42]	Chronic	B	16	BM	Intrathecal	4.8 × 10^7^	1	No AE	2/16 patients showed improvement in motor power of the upper extremities. Study was prematurely terminated due to poor results
[43]	Subacute	A, B and C	5	BM	Intrathecal	3–5 × 10^7^ BMSCs	1	No AE	Significant improvement in patients with AIS grade B and C only
[44]	Chronic	A and B	70	BM	Intrathecal	2 × 10^6^ BMSCs/kg b.w.	1–8 (monthly)	No AE	17/50 patients treated with cell therapy + physiotherapy showed improvement in AIS grade. 0/20 patients received physiotherapy only exhibited changes in AIS grade
[45]	Subacute	A	31	BM	Intrathecal	7 × 10^5^ to 1.2 × 10^6^ BMSCs	1	No AE	5/11 (45%) patients received BMSCs showed improvement in AIS grade from A to C compared to 3/20 (15%) patients in the control group
[46]	Subacute and chronic	A and C	30	BM	Intrathecal	1 × 10^6^ BMSCs/kg b.w.	2–3 (weekly)	No AE	No changes in AIS grade. Variable pattern of recovery in Barthel Index. No significant changes in SEP, MEP and NCV
[47]	Chronic	A, B, C and D	11	BM	Intrathecal	3 × 10^8^ BMSCs	3 (every 3 months)	No AE	Improvement in ASIA pin prick score, ASIA light touch score, ASIA motor score, IANR-SCIFRS score, VAS score and bladder function
[48]	Chronic	A	14	BM	Intraspinal	5 × 10^6^ BMSCs/cm^3^ lesion volume	1	No AE	7/12 patients showed improvement in AIS grade
[49]	Chronic	A	40	BM	Intraspinal	2 × 10^7^ BMSCs	1	No AE	9/20 patients showed improvement in AIS grade from A to B
[50]	Chronic	A, B and C	20	BM	Intraspinal	1 × 10^8^ BMSCs	1	No AE	4/8 (50%), 3/4 (75%) and 8/8 (100%) patients with AIS grade A, B and C respectively showed improvement in AIS grade
[51]	Subacute and chronic	A, B and C	20	BM	Intra-arterial and intravenous	104 ± 44.3 × 10^8^ BMMCs	1	No AE	Improvement in ASIA score in patients who received the cell therapy at the subacute phase and transplantation close to the lesion site (through arteria vertebralis)
[52]	Acute and chronic	A, B and C	8	BM	Intraspinal, intrathecal and intravenous	4 × 10^8^ BMMCs	1	No AE	6/8 patients showed improvement in AIS grade. The patients also showed improvement in Frankel grade, Ashworth score and ASIA score
[53]	Subacute	A	1	BM	Intrathecal, intravenous	3.7 × 10^9^ BMNCs & 1.54 × 10^8^ BMSCs	6 (every 3–4 months)	No AE	Improvement in AIS grade to C/D
[54]	Chronic	–	56	BM	Intrathecal	1 × 10^6^ BMNCs/kg b.w.	1	No AE	4 patients showed improvement in AIS grade and 24 patients have improved FIM score

*MSCs* Mesenchymal stem cells, *AIS* ASIA Impairment Scale, *BM* bone marrow, *AE* adverse event, *EMG* electromyography, *SEP* somatosensory evoked potential, *ASIA* American Spinal Injury Association, *MRI* magnetic resonance imaging, *BMMCs* bone marrow mononuclear cells, *BMNCs* bone marrow nucleated cells, *FIM* Functional Independence Measure, *MEP* motor-evoked potentials, *NCV* nerve conduction velocity, *IANR*-*SCIFRS* International Association of Neurorestoratology Spinal Cord Injury Functional Rating Scale, *VAS* Visual Analogue Scale

Most of the studies administered BMSCs intrathecally to the patients. Jeon et al. performed a phase I trial that administered BMSCs into the intramedullary space (8 × 10^6^ cells) and intradural space (4 × 10^7^ cells) of 10 patients at 1 month to 108 months post-SCI and more cells (5 × 10^7^ cells) were administered at 4 weeks and 8 weeks after the first cell transplantation through lumbar tapping [40]. It was found that the patients ASIA score, electromyography (EMG) and somatosensory evoked potential (SEP) improved after the treatment. Long-term follow-up of the patients showed that 3 patients with AIS grade B have better motor power of the upper extremities and activities of daily living as well as electrophysiological improvement. MRI demonstrated a reduction in lesion size and presence of fiber-like low signal intensity streaks [41]. The subsequent phase III trial was conducted with a modified protocol that doubled the number of cells administered into the intramedullary space (1.6 × 10^7^ cells), slightly reduced the amount of cell transplanted into the intradural space (3.2 × 10^7^ cells) and removed the second and third MSC transplantation at 4 weeks and 8 weeks [42]. Unfortunately, the study was prematurely terminated due to unexpected poor results whereby only 2 out of 16 patients showed improvement in motor power of the upper extremities. In another trial, Saito et al. [43] reported minimal improvement in the 3 patients with AIS grade A but significant improvement was observed in the 2 patients with AIS grade B and C after BMSC therapy. El-Kheir et al. [44] recorded an improvement in AIS grade in 17/50 patients treated with BMSCs and physiotherapy while none of the 20 patients treated with physiotherapy alone demonstrated AIS grade conversion. Karamouzine et al. [45] administered BMSCs into 11 patients with AIS grade A and found that 5/11 patients have their AIS grade improved to C, which is much better than the 3/20 recorded in the control group. Pal et al. [46] treated 30 patients with BMSCs but did not found conversion in AIS grade in any of the patients. Besides, there were also no significant changes in SEP, motor evoked potentials (MEP) and nerve conduction velocity (NCV) as well as a variable pattern of recovery in Barthel Index. Vaquero et al. [47] reported improvement in sensation, motor and bladder function, and IANR-SCIFRS score as well as a reduction in neuropathic pain in the chronic SCI patients (time of injury to participation 13.65 ± 14.79 years) received the BMSC therapy.

Several studies reported improvement in AIS grade in the chronic SCI patients that received the BMSCs through the intraspinal route. Mendonca et al. [48] found improvement in AIS grade from A to B in 6/12 patients and from A to C in one patient. Similarly, Dai et al. [49] found that BMSC treatment improved the AIS grade of 9/20 SCI patients from A to B while none of the 20 patients in the control group showed conversion in AIS grade. Jiang et al. [50] found that 8/8 (100%) patients with AIS grade C showed improvement in AIS grade after BMSC therapy compared to 3/4 (75%) and 4/8 (50%) patients with AIS grade B and A, respectively.

Apart from BMSCs, several studies applied the bone marrow mononuclear cells (BMMCs) and bone marrow nucleated cells (BMNCs) to treat SCI. Syková et al. treated 20 SCI patients with BMMCs and found that 5/6 patients received the cells close to the lesion site (through arteria vertebralis) showed improvement in ASIA score compared to only 1/14 patients that demonstrated ASIA score improvement after received the cells intravenously [51]. Besides, the authors also reported the benefit (improvement in ASIA score) of applying the cells during the subacute phase compared to the chronic phase. Geffner et al. [52] administered BMMCs through the intraspinal, intrathecal and intravenous routes to the same patients and reported improvement in AIS grade in 6/8 patients. Jarocha et al. [53] reported the transplantation of BMNCs at 10 weeks after SCI and subsequent transplantation of autologous BMSCs every 3–4 months for 5 times on a 15 years old patient with AIS grade A. On 2-year follow-up, the AIS grade improved to C/D (score increased from 112 to 231). Encouragingly, the patient’s control of body trunk, bladder filling sensation, bladder control, and anal sensation was restored, and muscle strength in lower extremities improved from plegia to deep paresis. Moreover, the patient regained the ability to stand and walk with support. Sharma et al. [54] transplanted BMNCs to 56 patients with chronic SCI (mean duration of injury 64 months) and found that 4 patients showed improvement in AIS grade and 24 patients have improved Functional Independence Measure (FIM) score.

### Umbilical cord-derived mesenchymal stem cells

UC-MSCs were used for the treatment of SCI due to its ease of sourcing, excellent in vitro expansion capacity, fast proliferation and low immunogenicity. UC-MSCs have low expression of HLA-ABC and no expression of HLA-DR and co-stimulatory antigens such as CD40, CD80, and CD86 that are associated with immune rejection [33, 55]. Additionally, UC-MSCs have more robust growth and more potent immune suppression property compared to BMSCs. Similar to BMSCs, UC-MSCs can be differentiated into multiple cell types, including the neural-like and glial-like cells [56–59]. The collection of UC-MSCs is noninvasive and its usage has not been encumbered with ethical issues as the cells are derived from redundant tissues and cause no harm to the donor. Although UC-MSCs are allogeneic, these cells utilize several mechanisms, including low expression of alloantigen, modulation of dendritic cell and T cell functions, and induction of regulatory T-cells to avoid immune rejection [60]. Table 3 shows the clinical trials conducted using UC-MSCs for the treatment of SCI. Liu et al. [7] reported the treatment of 22 SCI patients (time of injury to participation 2–204 months) with UC-MSCs and found that 13/22 patients showed improvement in ASIA score and IANR-SCIFRS score. However, this was only limited to the patients with incomplete SCI as none of the 6 patients with complete SCI showed improvement. They concluded that intrathecal injection of UC-MSCs can improve the neurologic function of chronic phase SCI patients with incomplete injury but not those with complete SCI. Cheng et al. [61] injected UC-MSCs intraspinally to 34 chronic phase SCI patients (time from injury to participation 12–72 months) with AIS grade A. They found that 7/10 patients received cell therapy demonstrated improvement in sensation, motion, muscle tension and self-care ability, whereas only 5/14 patients in the rehabilitation group and 0/10 patients in the untreated control group showed improvement. Besides, the UC-MSC treated patients also showed significant improvement in maximum bladder capacity and maximum detrusor pressure. In contrast, all the urodynamic indicators deteriorated among the patients in the rehabilitation group. The contradicting results between these 2 studies regarding the efficacy of UC-MSCs in amelioration of complete SCI may be due to the different route of cell transplantation as Liu et al. and Cheng et al. used intrathecal and intraspinal injection, respectively (Table 4).

**Table 3:** Clinical trials conducted using umbilical cord-derived mesenchymal stem cells for the treatment of SCI

References	Acute, subactute or chronic	AIS grade	Number of patients	Sources of MSCs	Route of administration	Number of cells	Frequency of injection	Safety outcome	Efficacy outcome
[7]	Chronic	–	22	UC	Intrathecal	1 × 10^6^ UC-MSCs/kg b.w.	Weekly 4 times as a course (17, 4 and 1 patients received 1, 2 and 3 courses respectively)	No AE	13/16 patients with incomplete SCI showed improvement in ASIA and IANR-SCIFRS score. 0/6 patients with complete SCI showed improvement
[61]	Chronic	A	34	UC	Intraspinal	4 × 10^7^ UC-MSCs	2 (separate by 10 days)	No AE	7/10 (70%), 5/14 (36%) and 0/10 (0%) patients in the UC-MSC, rehabilitation and untreated control group showed improvement in ASIA score, Barthel Index and muscle tension

*MSCs* Mesenchymal stem cells, *AIS* ASIA Impairment Scale, *UC* umbilical cord, *ASIA* American Spinal Injury Association, *IANR*-*SCIFRS* International Association of Neurorestoratology Spinal Cord Injury Functional Rating Scale, *AE* adverse event

**Table 4:** Clinical trials conducted using adipose-derived mesenchymal stem cells for the treatment of SCI

References	Acute, subactute or chronic	AIS grade	Number of patients	Sources of MSCs	Route of administration	Number of cells	Frequency of injection	Safety outcome	Efficacy outcome
[64]	Chronic	A, B and D	14	AT	Intrathecal	9 × 10^7^ ADSCs	3 (monthly)	No AE	10 patients have improved ASIA sensory score, 5 patients have improved ASIA motor score and 2 patients have improved voluntary anal contraction
[65]	Chronic	A	1	AT	Intrathecal	1 × 10^8^ ADSCs	1	No AE	The patient showed improvement in ASIA motor and sensory score as well as better quality of life as indicated by the higher Global Health Score.

*MSCs* Mesenchymal stem cells, *AIS* ASIA Impairment Scale, *AT* adipose tissue, *ASIA* American Spinal Injury Association, *AE* adverse event

### Adipose tissue-derived mesenchymal stem cells

Adipose tissue is ubiquitously available, can be easily collected using minimally invasive techniques such as subcutaneous lipoaspiration and contains a greater population of somatic stem cells compared to bone marrow [62, 63]. All these features render adipose tissue an ideal source of MSCs. Safety and efficacy of ADSC therapy in ameliorating SCI have been evaluated in a few human trials. Hur et al. [64] investigated the effect of intrathecal transplantation of autologous ADSCs in 14 patients with SCI. The result showed that 10 patients exhibited sensory improvement, 5 patients exhibited motor function improvement and 2 patients have improved voluntary anal contraction. However, MRI examination indicated that the lesion size remained unchanged. In a recent paper, Bydon et al. [65] reported the treatment of a SCI patient with 100 million autologous ADSCs. The cells were administered intrathecally. The patient showed improvement in ASIA motor and sensory score as well as improvement in the quality of life as indicated by the higher Global Health Score.

## Criteria to consider when applying mesenchymal stem cell therapy

**Fig. 4:**
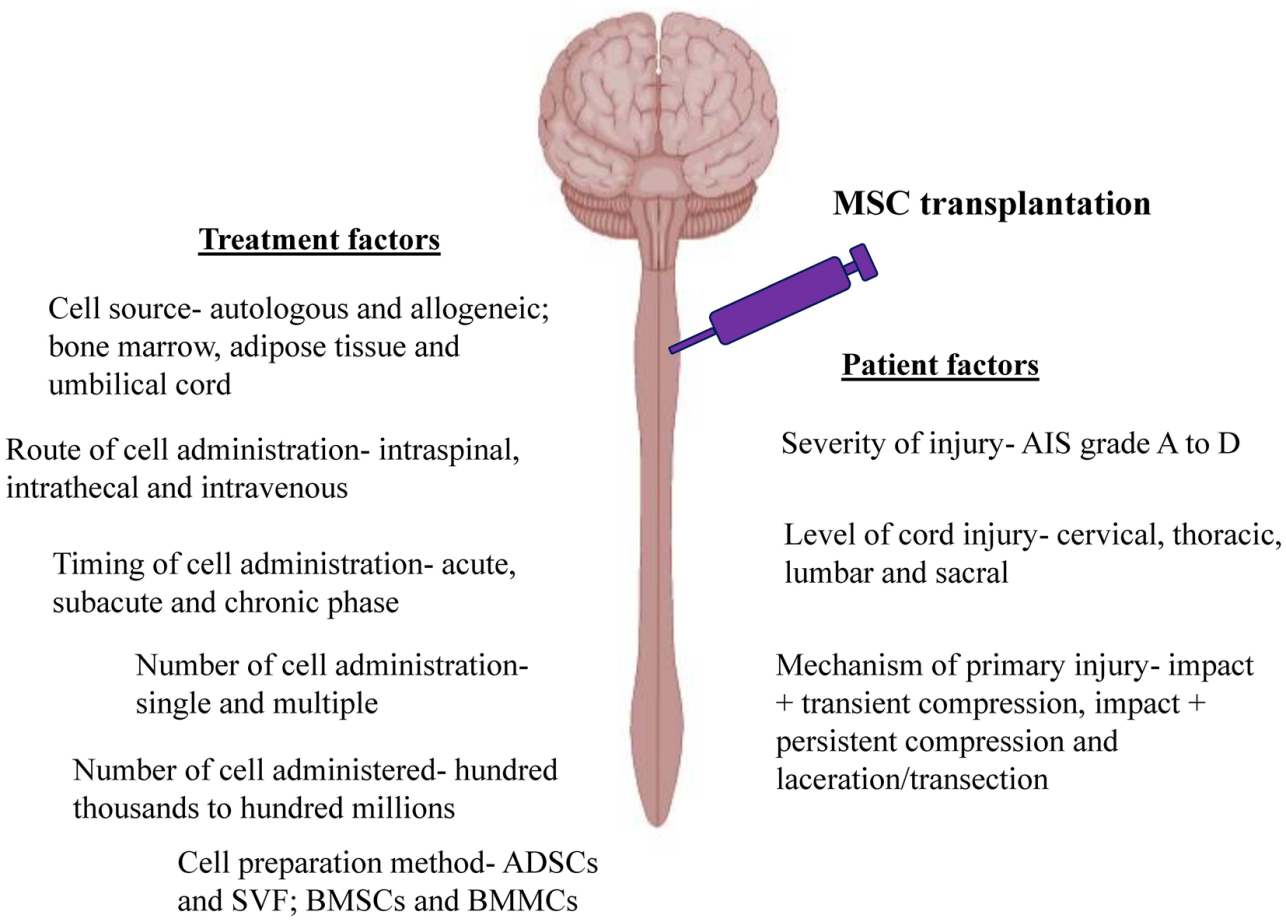
Factors affect the efficacy of MSC therapy to treat SCI. These factors can be categorized as patients factors that include severity of the injury, level of cord injury and mechanism of primary injury as well as treatment factors such as cell source, route of cell administration, timing of cell administration, number of cell administration, number of cells administered and cell preparation method. ADSCs—adipose tissue-derived mesenchymal stem cells, SVF—stromal vascular fraction, BMSCs—bone marrow-derived mesenchymal stem cells, BMMCs—bone marrow mononuclear cells, AIS—ASIA (American Spinal Injury Association) Impairment Scale

Based on the results reported in the clinical trials, MSC therapy is beneficial for SCI patients. However, not all the patients responded to the therapy and the improvement varied from patient to patient. These discrepancies can be due to patient variation, as they may have different severity of injury (AIS grade A to D), level of cord injury (cervical, thoracic, lumbar and sacral) and mechanism of primary injury (impact + transient compression, impact + persistent compression and laceration/transection). Apart from patient factors, variation in the MSC therapy such as cell source (allogeneic and autologous; bone marrow, umbilical cord and adipose tissue), route of cell administration (intrathecal, intraspinal and intravenous), timing of cell administration (acute, subacute and chronic phase), number of cell administration (single and multiple), number of cells administered (a few million to several hundred million) and cell preparation methods (BMSCs and BMMCs) also will affect the efficacy of the therapy (Fig. 4).

Both autologous and allogeneic MSCs can be used to treat SCI as the cells are immunoevasive. However, experiments on animal SCI models suggested that autologous MSCs are more effective compared to the allogeneic MSCs in treating SCI. ADSCs in the form of stromal vascular fraction (SVF) has been transplanted intraspinally to a SCI mouse model and the results showed that treatment with autologous SVF significantly improved the locomotor score compared to treatment with allogeneic SVF [66]. Besides, less demyelination and more integral structure were seen in spinal cord treated with autologous SVF. Similarly, autologous BMSCs transplanted intrathecally in a canine model were found to be more effective compared to allogeneic BMSCs in ameliorating SCI as indicated by the higher Olby score and smaller cystic cavity in the histological analysis [67]. The poorer efficacy of allogeneic MSCs is likely due to the more rapid clearance of the cells compared to the autologous counterpart.

MSCs from various tissue sources, including bone marrow, umbilical cord and adipose tissue, have been tested clinically for the treatment of SCI. However, none of the clinical studies compared the MSCs from different tissue sources. A previous study compared the efficacy of ADSCs and BMSCs in treating SCI using a mouse model found that both MSCs are equally potent in improving the animal motor function [68]. However, ADSCs have higher survival rate upon transplantation. More studies need to be conducted in future to determine the best source of MSCs to treat SCI. Table 5 shows the comparison between BMSCs, ADSCs and UC-MSCs for the treatment of SCI.

**Table 5:** Comparison between BMSCs, UC-MSCs and ADSCs for the treatment of SCI

Characteristics	BMSCs	UC-MSCs	ADSCs
Harvesting procedure	Invasive	None invasive	Invasive
Easiness to isolate and culture	Easy	Easy	Easy
Effect of age on cell quantity and quality	Quantity and quality decline with age	Unaffected	Quantity and quality decline with age
Potency to differential into nerve cells	Lower	Higher	Lower
Cell renewal	Lower proliferative potential	Higher proliferative potential	Lower proliferative potential
Expression of embryonic markers	Lower	Higher	Lower
Immune modulatory property	Good	Good	Good
Allogeneic cell rejection	No	No	No
Ethical issues	No	No	No
Risk of tumorigenicity	Very low	Very low	Very low

**Fig. 5:**
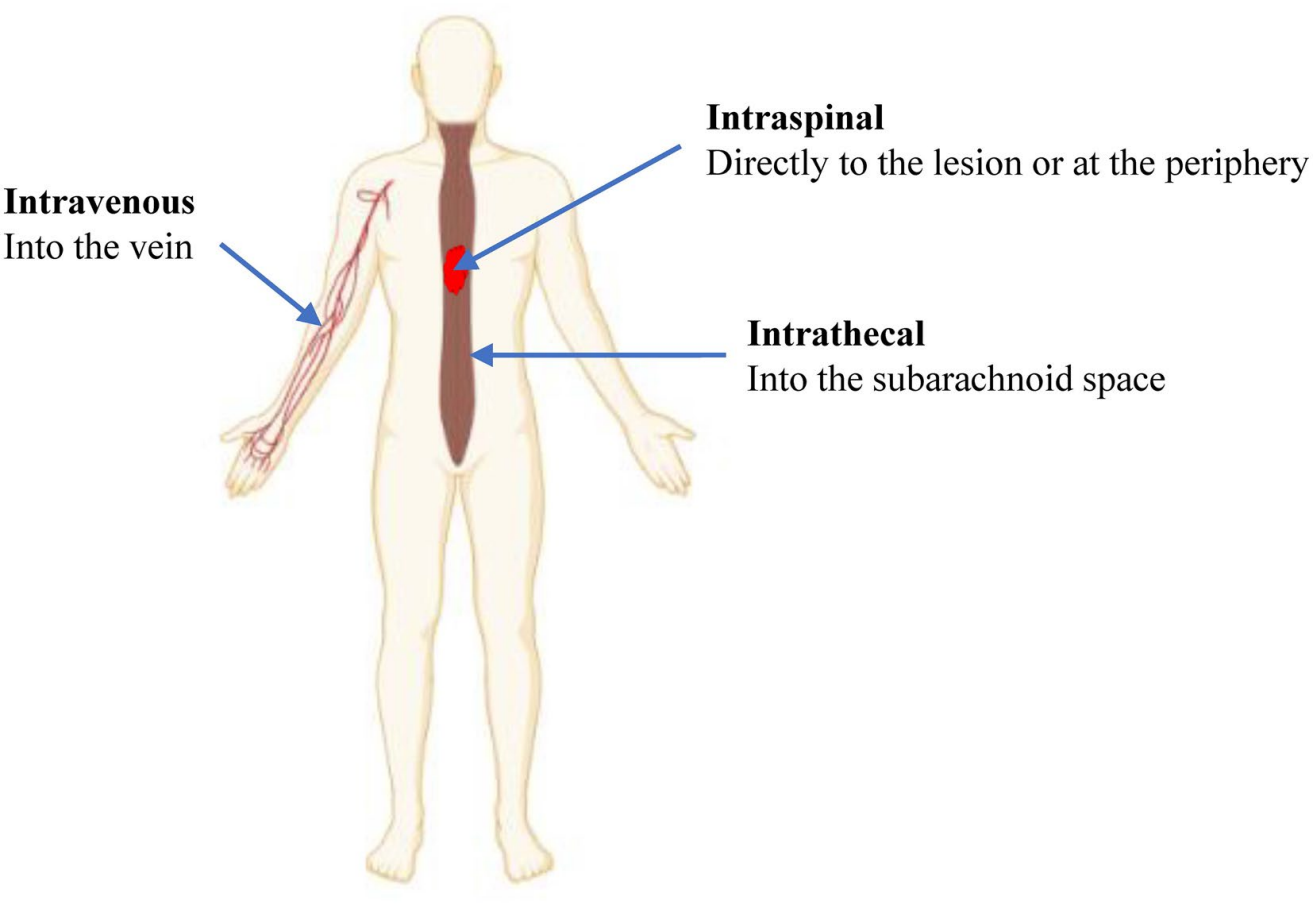
Routes of MSC administration. MSCs have been transplanted to the SCI patients via the intravenous, intraspinal and intrathecal route

MSCs have been administered via the intraspinal, intrathecal and intravenous route to the SCI patients (Fig. 5). The intraspinal route involves the direct transplantation of cells to the spinal cord, either directly on the lesion or at the periphery, while the intrathecal route involves the administration of cells into the subarachnoid space of the spinal cord. For the intravenous route, it is postulated that the transplanted MSCs will migrate across the BSCB to the spinal cord, attracted by the chemotactic factors at the lesion [69]. Most of the clinical studies administered the cells intraspinally and intrathecally because of the low migration of intravenously transplanted cells to the lesion [70]. Majority of the cells transplanted intravenously are trapped in the lung and other organs and only a small fraction of the transplanted cells will migrate to the lesion. Thus, a higher cell number is needed when the cells are administered intravenously. Syková et al. [51] reported improvement in ASIA score in 5/6 patients who received BMMCs near to the lesion (via the arteria vertebralis) while only 1/14 patients who got the cells intravenously showed improvement.

MSC transplantation can be performed during the acute, subacute (or intermediate) and chronic phase of SCI. Most clinicians consider the subacute phase as the optimal period for MSC therapy because the inflammatory process in acute phase will harm the injected cells while glial scar formed at the chronic stage inhibits axonal regeneration [71, 72]. A clinical study had shown that SCI patients who received MSC therapy during the subacute phase acquired greater improvement in ASIA score compared to those treated during the chronic phase [51].

Ideally, a single administration of MSCs is sufficient to exert its regenerative effect. However, a significant number of clinical studies administered the cells multiple times to get positive results [7, 40, 41, 44, 46, 47, 53, 61, 64]. It is postulated that the transplanted cells only can survive for a limited period in the hostile environment. Thus, extra injection is needed to deliver viable cells that can modulate the wound environment to favor regeneration. Worth mentioning is the study by a Korean group that found that reduction of MSC administration from 3 times in the preliminary study to 1 time in the phase III trial, despite increasing the number of cells transplanted, resulted in poorer therapeutic effect [41, 42]. Similarly, there is no consensus on the ideal number of cells for the treatment of SCI. In the clinical studies, the number of MSCs transplanted ranged from 7 × 10^5^ to 10.4 × 10^9^ cells [45, 51]. More studies are needed in future to determine if multiple infusions are beneficial for the treatment of SCI and to determine the ideal cell number to be transplanted.

Generally, MSC therapy appears to be more effective in patients with AIS grade B and C compared to those with AIS grade A [41, 43, 50]. One of the mechanisms of action of MSCs is via the secretion of paracrine factors which promote the survival and proliferation of remaining neurons. However, in the more severe AIS grade A, the number of preserved neurons is likely to be lower. Thus, it leads to poorer regeneration after MSC transplantation. In the future, more clinical trials with larger sample size are needed to support these findings and more preclinical studies are necessary to elucidate the mechanism involved.

## The future of MSC therapy for spinal cord injury

Clinical translation of MSC therapy in SCI progressed relatively slow despite encouraging results were reported in the preclinical studies on animal models. Currently, there is a lack of phase III trial exploring the therapeutic efficacy of MSC transplantation. This is caused by several reasons, including regulatory issues concerning the therapeutic application of MSCs, the high cost of phase III trial and low patient willingness to receive novel therapies without concrete evidence of clinical efficacy. Modest clinical efficacy and poor study design of the preliminary study also hindered the progression of MSC therapy. For example, a phase III study in Korea examining the effectiveness of BMSC therapy in 16 patients with chronic SCI reported that injection of 1.6 × 10^7^ and 3.2 × 10^7^ cells into the intramedullary and subdural space respectively resulted in poor therapeutic effect whereby only 2 patients showed neurological improvement [42]. The researchers of the aforementioned study used a different treatment protocol modified from the preliminary study due to regulatory requirements as multiple stem cell administration in phase III trial is typically prohibited by the authority [41]. Despite all the challenges, the future of MSC therapy for SCI remains bright as it has shown excellent efficacy in certain clinical studies.

## Novel biological therapies for spinal cord injury

Several new therapeutic adjuncts and treatment strategies which include stem cell secretory product-based therapy, scaffold-based therapy and immunotherapy may be used together with MSC therapy to enhance the therapeutic efficacy of MSC by improving the cells survival, migration, engraftment and proliferation.

### Stem cell secretome therapy

The application of MSCs to treat SCI showed encouraging result in both preclinical and clinical studies owing to its excellent immunomodulatory and regenerative capacities. However, the regenerative effects of stem cells do not solely rely on its differentiation potential and its ability to replace the damaged tissues, but also mediated by its secretome via the paracrine and autocrine mechanisms [73]. Stem cells derived-secretome is defined as the set of bioactive factors secreted to the extracellular space which comprise of soluble proteins, nucleic acids, lipids, cytokines, neurotrophins, growth factors and extracellular vesicles (EVs) [74]. Recently, there has been a paradigm shift from cellular therapy to secretome therapy due to several advantages of stem-cells derived secretomes, including its low tumorigenicity potential as secretomes do not endogenously replicate, scalable production, circulate for an extended period within the body, easily obtainable from the cell culture medium, modifiable bioactive content and can be stored without using toxic agents such as dimethyl sulfoxide (DMSO) [75, 76]. Many in vitro and preclinical studies have shown that MSCs-derived secretomes exert immunomodulatory, neurotrophic/neuroprotective and angiogenetic effects in various SCI models to promote nerve regeneration [69, 77].

Among various components of secretomes, EVs, composing of microvesicles and exosomes are considered to be the most valuable therapeutic agent [74]. MSC-EVs administered intravenously and intranasally in rats with SCI were found to accumulate and incorporated into the neurons at the lesion site [78, 79]. MSCs-derived EVs have been demonstrated to promote angiogenesis, suppress inflammation, reduce the lesion size, prevent neuronal cells apoptosis, support neuronal cells survival and regeneration, improve the integrity of BSCB and prevent glial scar formation, eventually improve the neuronal and motor functions using the in vitro and in vivo models [80–83]. Besides, MSC-EVs were found to be neuroprotective by reducing the A1 astrocytes that are neurotoxic to the synapse, myelin sheath and neurons through inhibition of NFκB p65 subunit nuclear translocation [84]. In the same study, the authors also reported that MSC-EVs were on par with MSCs in ameliorating SCI. Motor function recovery in SCI is often associated with the integrity of BSCB as disruption of BSCB and insufficient pericytes could hinder the recovery of nerve tissues. Recently, Lu et al. [81] demonstrated that MSC-EV administration suppressed the migration of pericytes and improved the integrity of the BSCB via down-regulation of NF-κB p65 signaling. Interestingly, EVs also could exert analgesic effects in a nerve injury-induced pain rat model apart from suppressing the inflammation and promoting the growth of neuronal cells [85].

Nowadays, EVs are also being explored as a biological vehicle to transfer bioactive factors to the damaged spinal cord. A previous study showed that miR-133b played a vital role in neuron differentiation and neurite outgrowth [86]. Hence, MSCs were modified to secrete miR-133b-rich EVs for the treatment of rats with SCI [87]. Treatment with miR-133b-rich EVs promoted the recovery of hindlimb locomotor function, reduced the lesion volume, protected the neuronal cells and enhanced axon regeneration compared to the EV and control groups. MSCs-derived exosomes also have been modified to transfer miR-29b and miR-216a-5p to promote regeneration of injured spinal cord [88, 89]. Interestingly, MSCs-derived exosomes also have been used to deliver phosphatase and tensin homology small interfering RNA (PTEN) that promote neuronal cell growth, support angiogenesis and suppressing gliosis when the exosomes were delivered intranasally to the rat SCI model [79].

There are several hurdles to overcome in the clinical application of secretomes/EVs. These obstacles include biodistribution of secretomes/EVs, persistency of secretome/EV biological content and unestablished regulatory requirement for the manufacturing and application of secretomes/EVs. However, as secretomes/EVs showed great promise as a therapeutic agent for SCI, secretome/EV treatment undoubtedly constitute a compelling approach for the treatment of SCI in the future.

### Immunotherapy

A major challenge in spinal cord regeneration is the presence of growth-suppressive molecules which create a post-trauma milieu that impedes healing. Despite the complexity of the inhibitory interactions, investigations have shown that the application of immunotherapy in acute SCI model could modulate the molecular signaling pathway and modify the injury site microenvironment to favour regeneration [90, 91]. Research conducted on a rodent SCI model indicated that antibodies targeting myelin-associated inhibitors could increase the locomotor function after injury [92]. Another study administered B-cell-depleting antibodies to control injury-associated inflammation and the treatment was found to subside the inflammation, promote cell survival and reduce hindlimb dysfunction [93]. Thus, the combination of immunotherapy and MSC treatment may be more effective in treating SCI by suppressing the inflammation and promoting cord regeneration. However, before these therapies can be used in patients, its safety must be clearly demonstrated by proving these antibodies do not affect healthy myelin and will not elicit detrimental immunological responses.

### Scaffolds

Scaffolds and injectable hydrogels are being explored as a matrix to promote cell engraftment and cell survival during transplantation. The scaffolds and hydrogels can be made of natural, synthetic or combined materials [94]. Previously, it has been reported that MSCs seeded in the fibrin scaffold showed good viability up to 10 weeks upon transplantation using the rat SCI model [95, 96]. With the advancement in biomaterial research, researchers have developed scaffolds that not only retain the cells at the lesion site and support cell survival but also modulate the cell functionality by enhancing its capability to reduce fibrosis and inflammation as well as to promote angiogenesis and neurogenesis. Furthermore, the scaffold itself also has the function of inhibiting glial scar formation, subside inflammation, stimulate angiogenesis and promote neurogenesis [94, 97]. The systematic review and meta-analysis conducted by Yousefifard et al. [98] showed that combination of MSCs and scaffolds is more effective compared to MSCs and scaffolds alone in improving the motor function in animal SCI models.

A recent study that reported a phase I clinical trial on 40 patients with acute complete SCI found that the patients received collagen scaffold + UC-MSCs demonstrated improvement in ASIA grade, activities of daily living (ADL) score, bowel and bladder function, and MEP compared to those in the control group without UC-MSCs-loaded scaffold that showed no improvement after 12 months [99]. MRI of the treated patients showed nerve regeneration and the diffusion tensor imaging illustrated that the electrophysiological activity was recovered. Another study by Zhao et al. [36] demonstrated that treatment with NeuroRegen, a collagen scaffold, with umbilical cord blood-derived MSCs (UCB-MSCs) in 8 patients with chronic complete SCI successfully enhanced the sensation level and increased the MEP-responsive area even though there was no change in ASIA grade. Results from these clinical studies clearly showed that scaffold can modulate the wound microenvironment to facilitate spinal cord regeneration.

Scaffolds have shown great promise in promoting regeneration of damaged spinal cord. However, several parameters, including biodegradation rate, biocompatibility, material safety and thickness of the matrix, need to be taken into consideration before clinical translation. In addition, issues such as product persistency and effectiveness also need to be resolved.

## Conclusion

MSCs from multiple sources, i.e. bone marrow, adipose tissue and umbilical cord, have been used clinically to treat SCI. Many of these trials were conducted on patients with chronic SCI that have been paralyzed by the disease for years. Generally, MSC therapy is safe but unable to improve the neurological function of all treated patients. Nonetheless, it shed light on the SCI patients that have failed all other therapies by helping them to regain some sensory and motor function. Further studies are underway to improve the delivery of MSCs and MSCs-derived products via scaffolds or by combining with immunotherapy to improve the treatment efficacy.

## Data Availability

Not applicable.
